# Spatial Differentiation and Driving Mechanisms of Nekton Community Diversity in Eastern Guangdong Coastal Waters, Northern South China Sea

**DOI:** 10.3390/biology15100768

**Published:** 2026-05-12

**Authors:** Yang Li, Mai Tong, Xi Zheng, Que-Hui Tang, Yan-Ping Zhang, Yu-Song Guo, Zhong-Duo Wang, Jian Liao

**Affiliations:** 1 Key Laboratory of Aquaculture in South China Sea for Aquatic Economic Animals of Guangdong Higher Education Institutes, College of Fisheries, Guangdong Ocean University, Zhanjiang 524088, China; liyang@stu.gdou.edu.cn (Y.L.); tm125@stu.gdou.edu.cn (M.T.); zx133@stu.gdou.edu.cn (X.Z.); zhangyanping6000@163.com (Y.-P.Z.); gysrabbit@163.com (Y.-S.G.); wangzd@gdou.edu.cn (Z.-D.W.); 2 South China Sea Fisheries Research Institute, Chinese Academy of Fishery Sciences, Guangzhou 510300, China; tangquehui@scsfri.ac.cn; 3 Guangdong Provincial Key Laboratory of Aquatic Animal Disease Control and Healthy Culture, College of Fisheries, Guangdong Ocean University, Zhanjiang 524088, China; 4 Key Laboratory of Marine Ecology and Aquaculture Environment of Zhanjiang, Guangdong Ocean University, Zhanjiang 524088, China

**Keywords:** Northern South China Sea, nekton, community, *Alpha* diversity, *Beta* diversity

## Abstract

Coastal waters of eastern Guangdong are important fishing grounds and ecologically sensitive areas in the northern South China Sea, where nekton communities are increasingly affected by environmental heterogeneity and human activities. However, systematic studies on the spatial differentiation and driving mechanisms of nekton communities in this region remain insufficient. This study aimed to clarify the community structure, diversity distribution characteristics, and key driving environmental factors of nekton in the coastal waters of eastern Guangdong, and thereby provide scientific support for an ecological health assessment and sustainable utilization of fishery resources in this region. Based on bottom-trawl survey data from 19 stations in the coastal waters of eastern Guangdong, northern South China Sea, this study systematically analyzed the species composition, dominant species, and diversity distribution pattern of nekton and their correlations with environmental factors using methods including the Index of Relative Importance, *Alpha* diversity indices, *Beta* diversity indices, and redundancy analysis. A total of 119 nekton species belonging to three phyla, four classes, 14 orders, and 56 families were collected. Among them, there were 79 fish species (accounting for 66.39%), 36 crustacean species (30.25%), and four cephalopod species (3.36%). The dominant species were *Trachypenaeus curvirostris* and *Portunus sanguinolentus* (*IRI* ≥ 1000). Wilcoxon’s test showed that there were significant differences in the Shannon–Wiener index, Gini–Simpson index, and Pielou’s evenness between the nearshore and offshore groups, while no significant regional difference was observed in the richness index. Cluster analysis, based on the Bray–Curtis distance, divided the 19 stations into five clusters, with significant differentiation in species composition and functional structure within the nearshore group. RDA results indicated that environmental factors collectively explained 99.66% of the variation in community structure. Particulate Inorganic Carbon (PIC), Phosphate (PO_4_^3−^), Distance to Port, Summer Maximum Chlorophyll-a (Chl-a), and Total Suspended Matter (TSM) were identified as the key driving factors. The coastal waters of eastern Guangdong boast rich nekton species, with significant differences in community structure between nearshore and offshore areas. The heterogeneity of the natural environment and human activity disturbances jointly shape the nekton diversity pattern in this region. The research results can provide a theoretical basis for regional marine ecological protection and fishery resource management.

## Simple Summary

Nekton are ecologically and economically essential to marine ecosystems, and their community diversity indicates marine ecological health. The eastern Guangdong coastal waters (northern South China Sea) lack systematic nekton diversity research, despite high biodiversity. This study surveyed 19 stations via bottom trawling, analyzing nekton community structure and environmental drivers with ecological methods. We collected 119 nekton species (fish-dominant), with *Trachypenaeus curvirostris* and *Portunus sanguinolentus* as dominant species. Nearshore–offshore communities differed in key diversity indices, with higher nearshore heterogeneity. PIC, phosphate, port distance, summer Chl-a, and TSM drove community variation (99.66%). Natural and anthropogenic factors shaped nekton diversity, with primary productivity amplifying spatial differences, filling regional research gaps, and supporting local marine conservation and fishery management.

## 1. Introduction

Nekton constitutes a key component of marine ecosystems and encompasses a variety of biological groups with significant ecological and economic value [1]. Its community structure and diversity not only directly reflect the health status of marine ecosystems but also serve as a critical foundation for the sustainable utilization of marine resources [2]. In recent years, driven by global climate change, overexploitation of marine resources, and the accelerated process of coastal industrialization, marine ecosystems have been subjected to multiple stressors, with ecological degradation being particularly severe in tropical and subtropical coastal waters [3]. As one of the important coastal waters in China, the eastern Guangdong coastal waters have become a research focus due to their high biodiversity and abundant fishery resources [4].

The eastern Guangdong coastal waters are located on the northern continental shelf of the South China Sea and in the coastal transition zone between Fujian and Guangdong Provinces, covering key ecological areas such as the Hanjiang River Estuary and the adjacent waters of Shantou Port [5]. Their unique geographical location and complex hydrological conditions (e.g., runoff input, water temperature and salinity gradients, and differences in sedimentary environments) provide habitats, spawning grounds, and foraging areas for a wide range of nekton species [4,6]. Meanwhile, this sea area is an important fishery operation zone along the southeast coast of China. Intensive nearshore aquaculture, busy port shipping, and frequent fishing activities are widely distributed in coastal zones, resulting in continuous anthropogenic disturbances that may affect the local nekton community structure [2,5]. The superposition of natural environmental heterogeneity and anthropogenic stressors may lead to significant spatiotemporal differentiation characteristics in the community structure and diversity patterns of nekton. However, existing studies have mostly focused on the resource assessment of single species or diversity surveys in local areas of the eastern Guangdong coastal waters [6,7,8], lacking a systematic analysis of the whole-area nekton community structure based on systematic survey data. Furthermore, the formation mechanism of diversity patterns driven by the combined effects of natural environmental factors and human activities (e.g., port disturbance and fishery pressure) remains unclear, which hinders the scientific support for an ecological health assessment and for refined fishery resource management in this sea area.

In terms of international research trends, a large number of existing studies have focused on the multiple impacts of climate change and human activities on marine ecosystems. For instance, climatic factors (e.g., sea surface temperature rise and ocean acidification) can directly or indirectly alter the structure and function of biological communities [9,10]. In addition, anthropogenic stressors caused by coastal development—such as pollution, habitat loss, and overfishing—have further exacerbated the potential risks to nekton diversity and ecological balance [6]. A comprehensive assessment of the effects of these driving factors on ecosystem dynamics not only helps to deepen the understanding of the maintenance mechanisms of marine biodiversity but also provides a scientific basis for regional sustainable management and policy formulation [11].

Based on the above, this study takes the eastern Guangdong coastal waters as the research area and relies on bottom-trawl survey data collected from 19 sampling stations in November 2022. By adopting quantitative approaches, including diversity index analysis, cluster analysis, and redundancy analysis (RDA)—which are effective in revealing community spatial patterns and driving mechanisms—we systematically investigate the species composition, dominant species characteristics, and Alpha/Beta diversity of nekton, identify the key environmental driving factors, and preliminarily reveal significant nearshore–offshore differentiation in nekton community structure.

The results of this study aim to fill the gap in the systematic research on nekton diversity in the eastern Guangdong coastal waters, reveal the community assembly mechanism under the combined effects of natural and anthropogenic factors, and provide scientific and theoretical support for the delimitation of ecological protection red lines, for the rational allocation of fishery resources, and for sustainable development of marine ecosystems in this sea area.

## 2. Materials and Methods

### 2.1. Sample Collection and Identification

**Figure 1:**
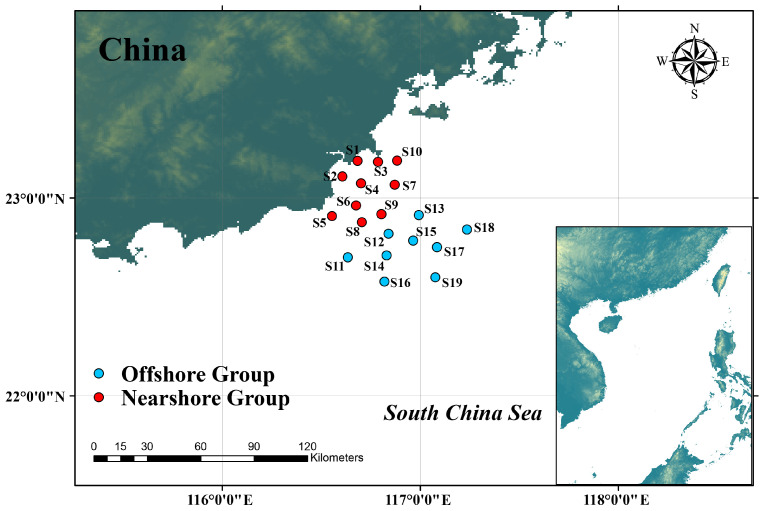
Distribution map of nekton sampling sites.

Nekton samples were collected in November 2022 from the eastern Guangdong coastal waters of the northern South China Sea (116.55° E~117.24° E, 22.58° N~23.19° N), where 19 sampling stations were set up for sample collection via bottom trawling (Figure 1). The field survey was conducted in November to avoid the fishing moratorium and typhoon season, and to protect fishery sustainability by staying outside the main fish spawning period. The sampling design was scientifically determined based on multiple key factors, including bathymetry and depth contours, salinity gradients, spatial distribution of the Hanjiang River Estuary, typical fishing ground distribution, and spatial heterogeneity of human activities (e.g., port shipping, coastal aquaculture, and industrial disturbance) in the study area. Stations were arranged to cover nearshore and offshore gradients, estuarine influence zones, traditional fishing grounds, and areas with different intensities of human disturbance so as to comprehensively represent the main habitat types and environmental heterogeneity of the eastern Guangdong coastal waters. The trawl net had a head rope length of 30 m, a net body length of 28 m, and a mesh size of 60 mm at the net mouth, with an average trawling speed of 2.9 knots during the survey. All surveys were conducted in the daytime. Considering a combination of environmental and vessel parameters, including trawling speed, trawling direction, current direction, current velocity, wind direction, and wind speed, the net was released at a distance of 2 to 3 nautical miles from the preset stations. The trawling speed was controlled at 2 to 3 knots with a trawling duration of 0.5 to 1 h, ensuring that the vessel arrived at or near the preset stations when trawling was completed. Based on the offshore distance of each station, the 19 sampling stations were divided into the nearshore group (10 stations) and the offshore group (9 stations). The taxonomic identification of the catches was conducted with reference to monographs, including *Marine Fishes of China* and *Fauna Sinica*, covering taxa such as fishes, shrimps, crabs, and cephalopods [12,13,14,15]. Distance to Port was defined as the shortest straight-line distance from each individual sampling station to the nearest major fishing port in the study area. This metric was calculated in ArcGIS 10.8. It serves as a proxy index to characterize the spatial gradient of fishing pressure, as fishing activities are inherently concentrated in proximity to ports with active harbor facilities. Fishery production data were obtained from the official website of the Shantou Municipal Bureau of Statistics (2022) (https://www.shantou.gov.cn/tjj/index.html (accessed on 1 April 2026)).

### 2.2. Data Analysis

#### 2.2.1. Determination of Dominant Species

Given the significant differences in the individual body size of the catches, the Index of Relative Importance (*IRI*) proposed by Pinkas et al. was adopted in this study to analyze the ecological status of each species caught in the quantitative composition of the nekton community, and the dominant species were identified accordingly [16]. The calculation formula of *IRI* is as follows:

*IRI* = (N + W) × F

where N = the percentage of individuals (ind) of a given species in the total number of individuals (ind) of the catches; W = the percentage of the weight of a given species in the total weight of the catches; and F = the percentage of the number of stations where a given species occurred in the total number of survey stations. Species with an *IRI* value greater than 1000 were defined as dominant species [17].

#### 2.2.2. *Alpha* Diversity Analysis

Four metrics, including the richness index, Shannon–Weiner index, Gini–Simpson index, and Pielou’s evenness index, were employed to analyze the *Alpha* diversity of the nekton community. The calculation formulas and definitions of each index are as follows: (1) Richness Index (*S*): This directly represents the number of distinct species in the survey sampling sites and reflects the basic species richness of the community composition. (2) Shannon–Weiner Index (*H′*): This index quantifies the level of species diversity in a community, with a higher value indicating greater species diversity [18]. Its calculation formula is:

$$H^{\prime }=-\sum_{i=1}^{S}P_{i}\ln P_{i}$$

where *p_i_* denotes the relative abundance of species i (the ratio of the individuals of a given species to the total number of individuals of all species). (3) Gini–Simpson Index (*D*): The classic Simpson index exhibits an inverse correlation with species richness, resulting in poor intuitive interpretation of the variation trend. Therefore, the derived Gini–Simpson index was adopted in this study to characterize community diversity. This index is calculated by subtracting the classic Simpson index from 1, and its value increases with rising species richness, showing a consistent variation trend with species richness [19]. Its calculation formula is:

$$D=1-\sum_{i=1}^{S}P_{i}^{2}$$

where *p_i_* is defined the same as above. (4) Pielou’s Evenness Index (*J*): This reflects the uniformity of the individual-number distribution among all species in a community, with a value range of 0 < *J*≤ 1. A value closer to 1 indicates a more uniform species distribution [20]. Its calculation formula is:

$$J=H^{\prime }/\ln(S)$$

For the diversity analysis, Wilcoxon’s test was performed on the *Alpha* diversity indices of nekton communities between the nearshore and offshore groups [21] to compare the differences in *Alpha* diversity of nekton communities between nearshore and offshore waters.

#### 2.2.3. *Beta* Diversity Analysis

Based on the catch-density data matrix of nekton from the 19 survey stations in the eastern Guangdong coastal waters, the Bray–Curtis dissimilarity matrix and Jaccard dissimilarity matrix were constructed, respectively, to quantify the differences in species composition of nekton communities among different survey stations via these two indices. All data analyses and graph plotting were conducted in R version 4.4.0. Specifically, the construction of dissimilarity matrices was implemented using the vegan package (v2.5-6) [22]; a clustered heatmap of Jaccard similarity coefficients was plotted using the online platform Hiplot (https://hiplot.cn (accessed on 1 April 2026)) to intuitively exhibit the similarity relationships of species composition among all stations; cluster analysis of the Bray–Curtis dissimilarity index was performed with the base stats package in R version 4.4.0 to further reveal the similarity and clustering characteristics of community structures across different stations.

#### 2.2.4. Relationship Between Nekton Community and Environmental Factors

Marine environmental data were obtained from the Global Marine Environment Dataset (GMED, https://gmed.auckland.ac.nz/index.html, accessed on 25 March 2026). The GMED is a gridded environmental dataset interpolated and simulated from long-term accumulated point-monitoring data, which has good universality and representativeness for regional ecological research. The spatial resolution of the GMED data used in this study is 30 arc-seconds, equivalent to approximately 0.926 km, which can effectively distinguish environmental heterogeneity among the 19 sampling stations. In ArcGIS 10.8, the latitude and longitude of the 19 stations were first converted into a point shapefile (*.shp*) layer. Using this point layer as a template, the Extract Multi Values to Points tool in the Spatial Analyst Tools (Arc Toolbox) was applied to extract environmental variables for each station. A total of 35 environmental factors were acquired (Table S1). For the 35 environmental factors across 19 stations, the Variance Inflation Factor (VIF) was used to test for multicollinearity, with VIF > 10 as the threshold for determining multicollinearity, and then highly correlated environmental factors were eliminated [23]. To improve the normality and homoscedasticity of the data, the remaining 5 environmental factors after screening were subjected to lg(x + 1) transformation [24]. To explore the response characteristics of the nekton community to environmental factors in the eastern Guangdong coastal waters, redundancy analysis (RDA) was performed on the processed data; prior to selecting the RDA model, detrended correspondence analysis (DCA) was conducted to determine the applicability of the analytical method. When the length of the first axis of DCA was less than 3, RDA was confirmed as the appropriate analysis method. All data analyses were completed using R software (Version 4.4.0).

## 3. Results

### 3.1. Species Composition

A total of 119 nekton species were captured in the survey of eastern Guangdong coastal waters, belonging to three phyla, four classes, 14 orders, and 56 families. The community composition exhibited distinct taxonomic dominance: fishes were the absolute dominant group, followed by crustaceans, and cephalopods with the least number of species, which constituted the main taxonomic structure of the nekton community in this sea area. Specifically, fishes accounted for the highest number of species (79 species), representing 66.39% of the total species; crustaceans ranked second with 36 species (30.25% of the total); cephalopods were the least diverse, with only four species (3.36% of the total). A detailed composition of the three major groups (fishes, crustaceans, and cephalopods) at the four taxonomic levels (order, family, genus, and species) is presented in Table 1.

**Figure 2:**
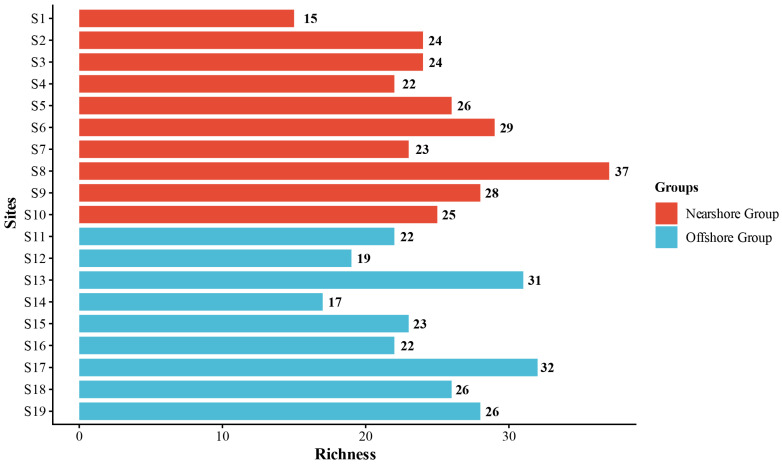
Species richness of nekton at 19 sampling sites in the eastern Guangdong coastal waters.

Among the 19 survey stations, significant spatial heterogeneity is observed in species richness: Station S6 had the highest species richness (38 species), while Station S1 had the lowest (15 species). The spatial distribution characteristics of species richness across all survey stations are illustrated in Figure 2.

### 3.2. Dominant Species

Analysis of the *IRI* for nekton dominant species in the eastern Guangdong coastal waters reveals that *Trachypenaeus curvirostris* (Hawk-claw shrimp, *IRI* = 1150.27) and *Portunus sanguinolentus* (Red-spotted swimming crab, *IRI* = 1118.72) are the primary dominant species in this sea area (Table 2). The catch abundance of *T. curvirostris* reached 830 individuals, accounting for 15.55% of the total abundance, with a catch weight of 6.055 kg, representing 4.32% of the total weight, making it the most ecologically important species overall. For *P. sanguinolentus*, the catch abundance was 226 individuals (4.23% of the total abundance), while the catch weight was 13.942 kg (9.94% of the total weight), ranking second only to *T. curvirostris* in comprehensive importance. The high *IRI* values of the two species indicate their pivotal ecological status in the nekton community of the eastern Guangdong coastal waters. *T. curvirostris* was mainly distributed at Stations S4, S5, S6, S7, S8, S10, S11, S13, S16, S17, and S19. In contrast, *P. sanguinolentus* was distributed across most survey stations, including S1, S2, S3, S4, S5, S6, S7, S8, S9, S10, S11, S13, S15, S17, and S19.

### 3.3. Alpha Diversity

**Figure 3:**
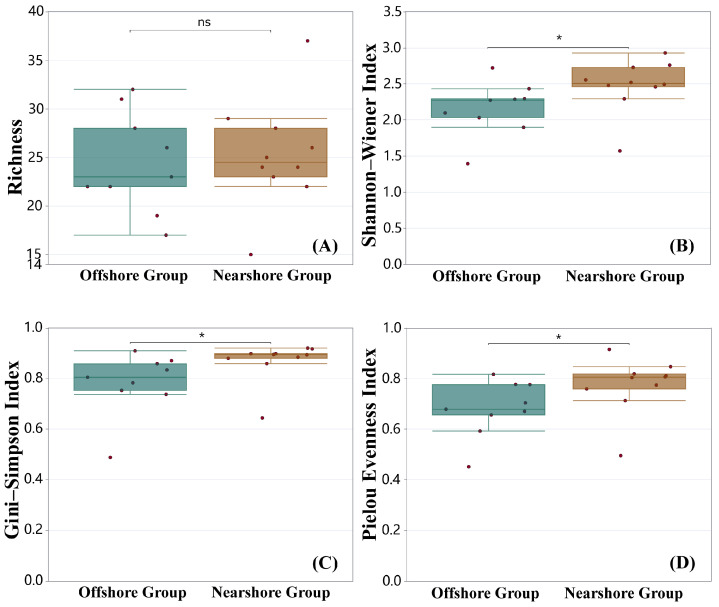
Comparative analysis of α-diversity indices of nekton between offshore and nearshore groups in the eastern Guangdong coastal waters. Note: ns = no significant difference; * = *p* < 0.05; dots represent individual samples.

Among the 19 survey stations (S1–S19) in this study, the distribution of *Alpha* diversity indices at each station is as follows: the richness index ranged from 15 (S1) to 37 (S8); the Shannon–Wiener index ranged from 1.393 (S11) to 2.928 (S8); the Gini–Simpson index ranged from 0.488 (S11) to 0.921 (S8); Pielou’s evenness index ranged from 0.451 (S11) to 0.915 (S1) (Table 3). Results of Wilcoxon’s test indicate that significant differences (*p* < 0.05) are detected in the Shannon–Wiener index, Gini–Simpson index, and Pielou’s evenness index between the nearshore group and offshore group, while no significant difference is observed in the richness index between the two groups (Figure 3).

### 3.4. Beta Diversity

**Figure 4:**
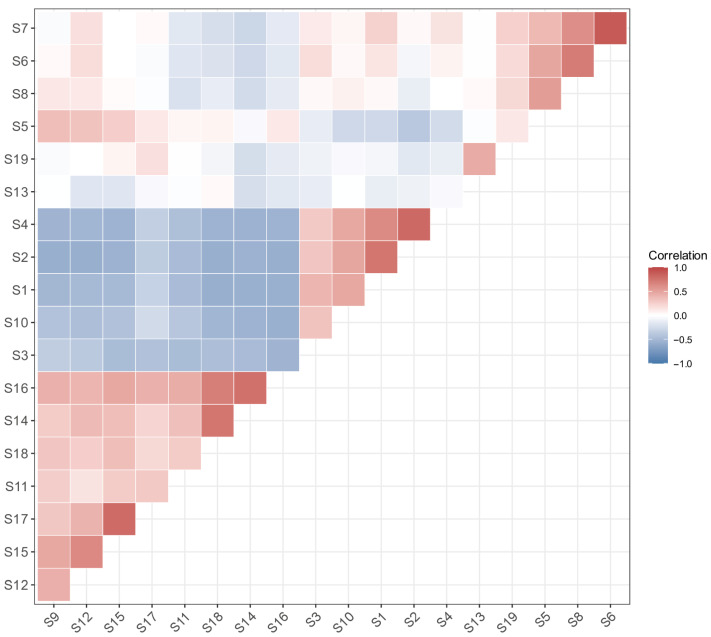
Jaccard index of nekton communities at 19 stations in the eastern Guangdong coastal waters.

Visualization results of associations based on the Jaccard index show (Figure 4) the highest species-composition similarity between Station S6 and Station S7 (Jaccard index = 0.733), followed by that between Station S15 and Station S17 (Jaccard index = 0.636). The lowest species-composition similarity is observed between Station S1 and Station S6, as well as between Station S8 and Station S9 (Jaccard index = 0.294 for both pairs).

**Figure 5:**
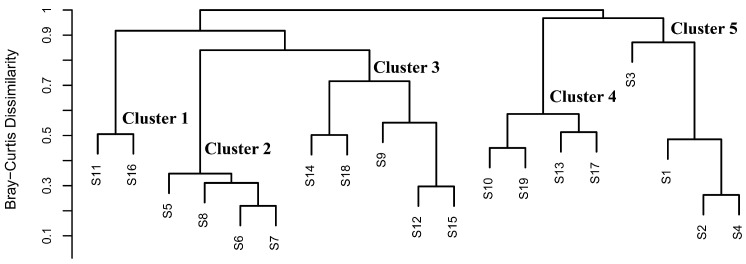
Clustering results of nekton communities at 19 sites in the eastern Guangdong coastal waters based on Bray–Curtis distance.

Results of the cluster analysis based on the Bray–Curtis dissimilarity coefficient (Figure 5) show that the nekton communities at the 19 survey stations in the eastern Guangdong coastal waters (S1–S10 for the nearshore group; S11–S19 for the offshore group) are clearly divided into five clustering clades (Cluster 1–Cluster 5) according to the similarity of species composition. The dissimilarity coefficients among the clades range approximately from 0.3 to 0.9, reflecting the degree of differentiation in community structure between different clades. The specific composition of each clade and its correlation with spatial grouping are as follows: Cluster 1 includes offshore group stations S11 and S16; Cluster 2 comprises nearshore group stations S5, S6, S7, and S8; Cluster 3 covers offshore group stations S12, S14, S15, and S18 and nearshore group station S9; Cluster 4 contains nearshore group station S10 and offshore group stations S13, S17, and S19; Cluster 5 consists of nearshore group stations S1, S2, and S4. Overall, the nearshore group stations (S1–S10) are scattered across four clades (Clusters 2, 3, 4, and 5) and do not form a concentrated and unified clustering unit, whereas the offshore group stations (S11–S19) are distributed across three clades (Clusters 1, 3, and 4), with relatively close clustering relationships among offshore stations within the same clade. Meanwhile, Cluster 3 and Cluster 4 exhibit the characteristic of mixed clustering of nearshore and offshore stations, indicating a certain similarity in the nekton community composition of some nearshore and offshore stations. In contrast, the scattered distribution pattern of nearshore stations reflects that their community-structure heterogeneity is significantly higher than that of the offshore stations, which may be related to the interference of multiple environmental factors in the nearshore area.

### 3.5. Relationships Between Nekton Communities and Environmental Factors

**Figure 6:**
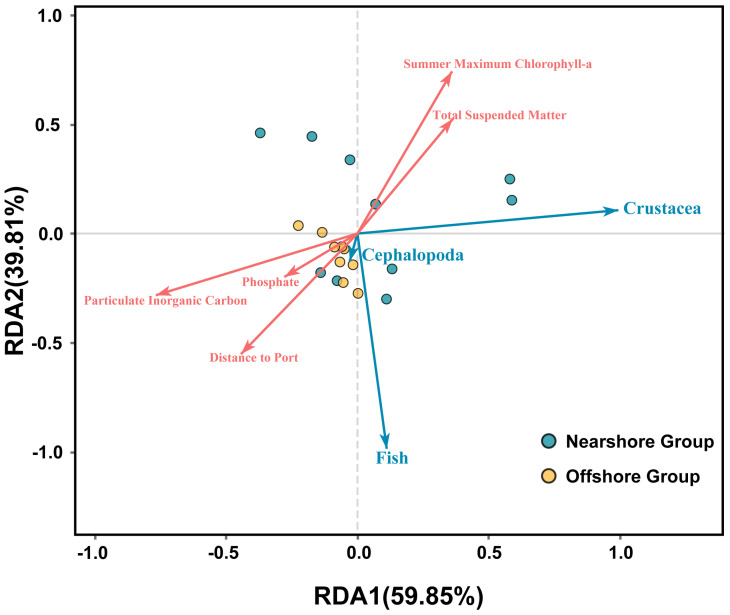
RDA analysis between nekton community and environmental factors in the eastern Guangdong coastal waters.

Results of the redundancy analysis (RDA) show (Figure 6) that the first two axes of RDA together explain 99.66% of the variation in nekton community composition in the eastern Guangdong coastal waters. Among them, the first axis (RDA1) accounts for 59.85% of the variation, and the second axis (RDA2) accounts for 39.81%, indicating that the ordination plot could well reflect the correlations between nekton communities and environmental factors. Particulate Inorganic Carbon, Phosphate, Distance to Port, Summer Maximum Chlorophyll-a, and Total Suspended Matter are identified as the key environmental factors affecting nekton community structure. Crustaceans are positively correlated with Summer Maximum Chlorophyll-a and Total Suspended Matter, while cephalopods and fishes are positively correlated with Particulate Inorganic Carbon, Phosphate, and Distance to Port. The nearshore group is mainly distributed in the positive regions of both RDA1 and RDA2 in the RDA ordination plot, whereas the offshore group is predominantly distributed in the negative regions of RDA1 and RDA2. Specifically, the environmental characteristics of the nearshore group have a stronger correlation with Total Suspended Matter, Summer Maximum Chlorophyll-a, and Phosphate, while the offshore group is more closely associated with Particulate Inorganic Carbon and Distance to Port. Such environmental heterogeneity is likely the key factor driving the spatial differentiation of nekton communities.

## 4. Discussion

### 4.1. Community Composition and Ecological Characteristics of Dominant Species

As an important component of the northern South China Sea, the coastal waters of eastern Guangdong serve not only as a maritime hub for external connectivity in eastern Guangdong but also as a key regional natural fishery ground and shipping lane, exerting significant significance for the development of Guangdong Province’s marine economy [25]. A survey was conducted at 19 stations in this sea area (adjacent to the Zhelin Bay Marine Ranch), and a total of 119 nekton species were identified, belonging to three phyla, four classes, 14 orders, and 56 families. The taxonomic composition exhibited a distinct dominance pattern: fishes were the most dominant group, accounting for 66.39% of the total species (79 species). Among them, 36 species belonged to Perciformes, representing 45.57% of the total fish species, which highlights their broad adaptability to water temperature, salinity, and habitat conditions in the coastal waters of South China. This finding is consistent with the research results of Liu et al. (2021) in the Zhanjiang Harbor waters. Crustaceans accounted for 30.25% of the total species (36 species), while cephalopods were the least dominant group, with only four species (3.36% of the total). Overall, this composition pattern is consistent with the taxonomic distribution regularity in the continental shelf waters of the northern South China Sea [26,27].

Artificial reefs deployed in the Zhelin Bay Marine Ranch provide shelters, foraging grounds, spawning grounds, and nursery grounds for fishes, laying a solid foundation for the growth of fish populations [27], and further consolidating the dominant position of fishes in the community. Based on the present trawl survey data, the lowest proportion of cephalopods may be closely related to the average nearshore water temperature being consistently above 20 °C during the survey period (November)—cephalopod growth and development are highly dependent on marine environmental temperature, and excessively high water temperatures can reduce their embryo survival rate and larval-to-adult survival rate [28], which aligns with the research viewpoint of Lu et al. (2024) that cephalopod growth relies on suitable temperatures.

Based on the present survey data, the dominant species in the study area were T. curvirostris (*IRI* = 1150.27) and P. sanguinolentus (*IRI* = 1118.72). Both species are distributed in most stations and exhibit prominent comprehensive importance. This result is closely associated with the abundant plankton resources in the nearshore area: surface runoff input from the Hanjiang River and other sources, together with upwelling, brings substantial salts and nutrients to the eastern Guangdong coastal waters [25,29], nourishing a large quantity of plankton. This provides sufficient bait for the filter-feeding *T. curvirostris* and benthic predatory *P. sanguinolentus* [30], enabling them to occupy core ecological niches in the community.

### 4.2. Spatial Differentiation of Diversity and Its Driving Mechanisms

Based on the survey data, results of the α diversity analysis showed that there were significant differences (*p* < 0.05) in the Shannon–Wiener index, Gini–Simpson index, and Pielou’s evenness index between the nearshore and offshore groups divided by distance from the shore, while no obvious regional differentiation was observed in the richness index. This special pattern is closely related to the strong mobility of nekton—species can perform short-distance migrations between nearshore and offshore areas within the survey station range, effectively maintaining the uniformity of regional species richness [31]. Among all survey stations, Station S8 had the highest Shannon–Wiener index (2.928) and Gini–Simpson index (0.921), indicating a high total number of species and rich community diversity at this station [18]. This result may be attributed to Station S8 being far from ports with less human activity interference; the stable habitat environment is more conducive to the growth, development, and reproduction of nekton [17].

The Jaccard index analysis showed that S6 and S7 in the nearshore group and S15 and S17 in the offshore group exhibited the highest and second-highest similarity, respectively (Jaccard indices of 0.733 and 0.636). This indicates that although the distance from the shore shapes the overall differences between nearshore and offshore communities through environmental factors, such as water depth and salinity [27], stations with consistent habitat conditions within the same group still maintain high species similarity. Notably, the species composition within the nearshore group showed significant differentiation, with the lowest Jaccard index (both 0.294) between S1 and S6, and between S8 and S9, resulting in low species similarity even within the nearshore area. This phenomenon is mainly related to the special environment of Station S1, as this station is adjacent to the Huaneng Haimen Power Plant [32], and the mechanical noise generated by the power plant has a repellent effect on sound-sensitive groups such as sciaenid fishes [33], prompting them to migrate to Station S6, where there is less noise interference. Ultimately, this led to higher species richness at Station S6 (38 species) than at Station S1 (15 species) (Figure 2). In addition, this study area is located in the coastal waters of Guangdong, where coastal mariculture activities are widely distributed. Escaped cultured fish may compete with wild nekton for food and habitat, thereby affecting the community structure of wild nekton, which is consistent with the RDA conclusion that “Distance to Port” serves as an indirect indicator of anthropogenic disturbance intensity. Port shipping, fishery harvesting, and aquaculture activities all affect community-composition heterogeneity by altering habitat environments or species migration patterns [6]. Fishes, crustaceans, and cephalopods are generally recognized as the core target species of fishery harvesting activities. Consequently, fishing pressure serves as a key driving factor regulating the community structure of nekton. As shown in Table 4, the main fishing targets of the marine fishery in the study area in 2022 included fishes, bivalves, and shrimps/crabs (a subset of crustaceans), and the relatively high fishing pressure has largely shaped and altered the distribution pattern of nekton in this region.

Results of the cluster analysis based on the Bray–Curtis dissimilarity coefficient showed that offshore stations were more closely clustered, while nearshore stations were scattered across four clades without forming a unified tight cluster (Figure 5), confirming that the community-structure heterogeneity in the nearshore area is significantly higher than that in the offshore area [24]. In addition to the aforementioned differences in species composition, this result is also closely related to differences in species-abundance structure and selective screening by environmental factors: Stations S1, S2, and S4 in Cluster 5 are significantly affected by noise from the Huaneng Haimen Power Plant [33]; noise disrupts the normal physiological activities of sensitive fishes, while some crustaceans show a certain tolerance to noise and can adapt to the environment by seeking shelters [34], which is consistent with the adaptive characteristics of crustaceans to the nearshore environment revealed by RDA. For the nearshore clade (S5, S6, S7, and S8) in Cluster 2, the nearshore area is more affected by river estuaries; Total Suspended Matter and Phosphate brought by river inflow [10,29] play a key role in shaping species-abundance structure. The synergistic effect of Total Suspended Matter and Phosphate significantly increases phytoplankton biomass [25,35], and Summer Maximum Chlorophyll-a—as a core indicator of primary productivity—directly determines the food availability for crustaceans [36]; thus, crustaceans are significantly positively correlated with Summer Maximum Chlorophyll-a and Total Suspended Matter. Meanwhile, suspended particulate matter also affects the euphotic depth of seawater, regulating plant photosynthetic efficiency [35] and indirectly influencing the distribution of zooplankton and nekton, ultimately leading to significant differences in species-abundance structure among different nearshore clades.

In contrast, fishes and cephalopods were positively correlated with Particulate Inorganic Carbon, Phosphate, and Distance to Port. Water depth is widely recognized as a key environmental factor influencing marine nekton communities. Although depth was included in our preliminary variable screening, it was excluded from further analysis due to a VIF value < 10, and thus was not identified as one of the primary drivers affecting nekton community structure in this study.

The low anthropogenic disturbance (longer Distance to Ports) and stable sedimentary environment (seabed substrate composed of Particulate Inorganic Carbon) in the offshore area [37] are more suitable for the survival of fishes and cephalopods, which have strong mobility and high requirements for habitat stability. Notably, the significant impact of Summer Maximum Chlorophyll-a on the autumn community reflects the seasonal cumulative effect of primary productivity—the vigorous growth of phytoplankton in summer provides a continuous food supply for subsequent nekton [25]. The distribution difference in Phosphate is mainly affected by Hanjiang River runoff input and marine aquaculture [10,29], and indirectly shapes the nekton food chain by regulating the plankton community, which is consistent with the conclusion of Zhang et al. (2022) that nutrients drive the differentiation of coastal biological communities.

Overall, the spatial differentiation of nekton diversity in the eastern Guangdong coastal waters is the result of the combined effects of natural environment, primary productivity processes, and human activities. Firstly, the distance from the shore shapes the overall differences between nearshore and offshore communities by regulating water depth, salinity, and sedimentary environment (e.g., Particulate Inorganic Carbon distribution), and stations with consistent habitats within the same group maintain high species similarity. Secondly, the coupling effect of nutrients and primary productivity is the core ecological process driving species-abundance differentiation—Phosphate and Total Suspended Matter directly affect the food availability of different groups by regulating phytoplankton biomass (characterized by Chlorophyll-a), further leading to the enrichment of crustaceans in the nearshore areas and the dominance of fishes and cephalopods in the offshore areas. Thirdly, the heterogeneity of human activities exacerbates the diversity differentiation in the nearshore area—port activities, industrial noise interference, and aquaculture escapes not only alter the local habitat environment but also change the community structure by selecting disturbance-tolerant groups (e.g., *T. curvirostris* and *P. sanguinolentus*), while the low anthropogenic disturbance in the offshore area maintains a more complex community composition. Fourthly, the seasonal cumulative effect of primary productivity further amplifies the spatial differences in community structure, reflecting the regulatory role of the temporal scale of ecological processes on spatial differentiation.

### 4.3. Limitations and Prospects

The study employed bottom trawling as the gear for nekton sampling. This method conforms to the standard protocol for demersal nekton community surveys in coastal, continental shelf, and flat-bottom areas—as recommended by the International Council for the Exploration of the Sea (ICES), the Common Fisheries Policy (CFP) of the European Union, as well as China’s Marine Monitoring Code and Fishery Resources Survey Code—and is highly compatible with the characteristics of the study area (shallow water depth, gentle bottom topography, and dominance of demersal and near-bottom nekton), representing the optimal choice matching our research objectives. Nevertheless, constrained by the inherent selectivity of sampling gear, bottom trawling has low capture efficiency for pelagic and mesopelagic nekton and cannot effectively cover the full nekton assemblage in the water column. Meanwhile, bottom trawling is restricted by habitat conditions and is difficult to deploy in complex terrains such as coral reefs, which may lead to an underrepresentation of the nekton communities in such habitats. Therefore, the results of this study mainly reflect the diversity characteristics of demersal and near-bottom nekton communities in the study area, and cannot fully represent the overall composition of nekton in the full water column and complex habitats. For future nekton research in this sea area, a combined sampling strategy of bottom trawling plus mid-water trawl or seine is recommended to achieve systematic sampling of nekton across the full water column and multiple habitats, to more comprehensively reveal the regional nekton diversity and its spatial distribution pattern. Furthermore, restricted by the insufficient long-term seasonal fishery monitoring records in the investigated waters, we failed to quantitatively analyze the seasonal dynamics and variation characteristics of local nekton assemblages, which should be supplemented in future long-term surveys. Although the GMED dataset has high universality and the 0.926 km resolution can effectively identify environmental differences among stations, global gridded data still have certain limitations. Gridded environmental data may not fully reflect the fine-scale local environmental conditions at the 19 sampling stations. In addition, the temporal resolution of GMED may not be completely synchronized with the one-time sampling in November 2022, which may lead to a slight spatiotemporal mismatch. Despite these constraints, the selected key environmental factors explained 99.66% of the community variation, indicating that the environmental data and analytical process were reliable and sufficient to reveal the spatial differentiation mechanisms of nekton communities in the study area.

## 5. Conclusions

The coastal waters of eastern Guangdong have long been affected by the dual superimposition of human activities (such as nearshore aquaculture, port disturbance, and fishing operations) and natural changes (such as ocean current dynamics and seasonal climate fluctuations), forming a complex and variable marine environment that directly shapes the habitat distribution and diversity characteristics of nekton in this sea area. This study aimed to characterize the community structure of nekton in the coastal waters of eastern Guangdong and to identify key environmental factors influencing their distribution. This study found that there are significant differences in the diversity patterns of nekton between nearshore and offshore areas. As core environmental factors, Particulate Inorganic Carbon, Phosphate, Distance to Port, Summer Maximum Chlorophyll-a, and Total Suspended Matter display differences across different distances from the shore, which directly regulate the species composition and diffusion processes of nekton, thereby resulting in obvious diversity differentiation in the study area. While these findings clarify the main nekton community patterns, this study was limited by insufficient seasonal data and certain constraints of GMED environmental data. Based on the above results, conducting long-term comprehensive monitoring of nekton diversity in the eastern Guangdong coastal waters can not only systematically clarify the evolution laws and key driving factors of nekton diversity patterns under the superimposition of natural environmental changes and human activities, but also provide a practical scientific basis for the protection and restoration of coastal ecosystems and the sustainable management of fishery resources.

## Figures and Tables

**Table 1:** Taxonomic status and species number of each nekton group at 19 sampling sites in the eastern Guangdong coastal waters.

Taxa	Order	Family	Genus	Species
Fishes	Myliobatiformes	2	2	2
	Orectolobiformes	1	1	1
	Gasterosteiformes	1	1	1
	Pleuronectiformes	4	7	9
	Clupeiformes	3	6	9
	Perciformes	20	31	36
	Anguilliformes	3	3	3
	Siluriformes	1	1	1
	Tetraodontiformes	2	3	6
	Aulopiformes	2	4	5
	Scorpaeniformes	3	6	6
Crustaceans	Stomatopoda	1	5	5
	Decapoda	11	18	31
Cephalopods	Octopoda	2	2	4
Total	14	56	90	119

**Table 2:** Catch parameters and *IRI* indices of dominant species of nekton at 19 sites in the eastern Guangdong coastal waters.

Species	Catch Abundance		Catch Weight		*IRI*
	(ind)	(%)	(kg)	(%)	
*T. curvirostris*	830	15.55	6.055	4.32	1150.27
*P. sanguinolentus*	226	4.23	13.942	9.94	1118.72

**Table 3:** *Alpha* diversity indices of nekton at 19 stations in the eastern Guangdong coastal waters.

Stations	Richness	*H′*	*D*	*J*
S1	15	2.478	0.898	0.915
S2	24	2.460	0.884	0.774
S3	24	1.572	0.644	0.495
S4	22	2.493	0.894	0.807
S5	26	2.759	0.917	0.847
S6	29	2.555	0.880	0.759
S7	23	2.520	0.895	0.804
S8	37	2.928	0.921	0.811
S9	28	2.727	0.898	0.818
S10	25	2.294	0.859	0.713
S11	22	1.393	0.488	0.451
S12	19	2.287	0.858	0.777
S13	31	2.033	0.753	0.592
S14	17	1.898	0.737	0.670
S15	23	2.432	0.871	0.776
S16	22	2.098	0.805	0.679
S17	32	2.273	0.783	0.656
S18	26	2.293	0.834	0.704
S19	28	2.721	0.910	0.817

Note: *H’* represents the Shannon–Wiener index, *D* represents the Gini–Simpson index, and *J* represents Pielou’s evenness index.

**Table 4:** Fishery production by districts and counties in Shantou, eastern South China Sea (2022).

Indicator	Shantou City	Jinping District	Longhu District	Chenghai District	Haojiang District	Chaoyang District	Chaonan District	Nan’ao County
Total Aquatic Products Output	474,439	17,620	9803	83,722	50,251	97,752	22,881	192,410
Marine Products	378,341	9820	3840	45,170	47,334	73,187	8710	190,280
Fish	156,352	1001	2084	21,134	26,289	35,631	5256	64,957
Shrimp and Crabs	76,559	2003	637	18,606	10,456	27,167	3410	14,280
Shellfish	84,092	6790	1119	3390	8025	8802	44	55,922

Note: data sourced from the official website of Shantou Municipal Bureau of Statistics (unit: ton) at https://www.shantou.gov.cn/tjj/index.html, (accessed on 13 April 2026.)

## Data Availability

Data will be made available upon request.

## Supplementary Materials

The following supporting information can be downloaded at: https://www.mdpi.com/article/10.3390/biology15100768/s1, Table S1: The marine environmental variables involved in this study; Table S2: Nekton species composition at each sampling station.

## Abbreviations

The following abbreviations are used in this manuscript:

PIC	Particulate Inorganic Carbon
PO_4_^3−^	Phosphate
Chl-a	Chlorophyll-a
TSM	Total Suspended Matter
RDA	Redundancy analysis
IRI	Index of Relative Importance
VIF	Variance Inflation Factor
